# Association of Polycystic Ovary Syndrome Phenotypes With Adverse Pregnancy Outcomes After *In-Vitro* Fertilization/Intracytoplasmic Sperm Injection

**DOI:** 10.3389/fendo.2022.889029

**Published:** 2022-06-03

**Authors:** Qiumin Wang, Honghong Wang, Ping Li, Xiufang Li, Ze Wang, Lei Yan, Yuhua Shi

**Affiliations:** ^1^ Center for Reproductive Medicine, Shandong University, Jinan, China; ^2^ Shandong Provincial Clinical Medicine Research Center for Reproductive Health, Shandong University, Jinan, China; ^3^ Children’s Hospital of Shanxi and Women Health Center of Shanxi, Taiyuan, China; ^4^ Women and Children’s Hospital, School of Medicine, Xiamen University, Xiamen, China; ^5^ Guangdong Provincial People’s Hospital, Guangdong Academy of Medical Sciences, Guangzhou, China

**Keywords:** polycystic ovarian syndrome, phenotype, assisted reproductive technology, hypertensive disorder of pregnancy, adverse pregnancy outcomes

## Abstract

**Objective:**

This study aims to evaluate the association between polycystic ovary syndrome (PCOS) phenotypes and adverse perinatal outcomes, comparing the characteristics, ovarian response, and assisted reproductive outcomes in patients with various PCOS phenotypes after *in-vitro* fertilization (IVF)/intracytoplasmic sperm injection (ICSI).

**Methods:**

This study comprised 6,732 patients who underwent the first cycle of IVF/ICSI treatment in our outpatient department from January 2017 to July 2018. Propensity score matching (PSM) was used in PCOS and non-PCOS groups to balance the influence of intergroup confounding factors. After the PSM procedure, 1,186 patients were included in the two groups, and the PCOS patients were further divided into four PCOS phenotype groups based on the Rotterdam criteria.

**Results:**

Patients with various PCOS phenotypes had similar rates of biochemical pregnancy, clinical pregnancy, and live birth (all *P*-values > 0.05). The overall incidence of adverse pregnancy outcomes (including ectopic pregnancy, miscarriage, preterm birth) was significantly higher in PCOS phenotype A and D groups than in the control group (44% and 46.4% vs. 28.7%, *P* = 0.027). The rates of hypertensive disorder of pregnancy (HDP) were significantly higher in PCOS phenotype A and C groups than in the control group (9.3% and 12.5% vs. 3.1%, *P* = 0.037). After adjustment for potential confounders, the differences in adverse pregnancy outcomes persisted (*P* = 0.025).

**Conclusions:**

The overall incidence of adverse pregnancy outcomes is higher in women with PCOS phenotypes A and D than in women with non-PCOS.

## Introduction

Polycystic ovary syndrome (PCOS) is a common endocrine disorder in women of reproductive age and the main cause of anovulatory infertility (1–3), which is characterized by obesity, hyperandrogenism, anovulation, insulin resistance, polycystic ovary, and infertility. The global prevalence of PCOS ranges from 6% to 21% (4); however, the etiology of PCOS is unclear (5). Moreover, because anovulation in women with PCOS often results in infertility (6), assisted reproductive technology (ART) is usually required for these women to become pregnant. According to the Rotterdam criteria, PCOS patients can be divided into the following four phenotypes: phenotype A—coexistence of clinical hyperandrogenism/hyperandrogenemia, oligomenorrhea/anovulation, and polycystic ovaries (HA+OA+PCO); phenotype B—clinical hyperandrogenism or hyperandrogenemia and oligomenorrhea/anovulation (HA+OA); phenotype C—clinical hyperandrogenism or hyperandrogenemia and polycystic ovaries (HA+PCO); and phenotype D: oligomenorrhea/anovulation and polycystic ovaries (OA+PCO) (7). For different PCOS phenotypes, the ovarian response to gonadotropin (Gn) is varied in controlled ovarian hyperstimulation (COH) (8), which in turn affects the outcome of ART.

Because PCOS patients have the characteristics of reproductive endocrine dysfunction and metabolic disorder (9), they were more prone to having pregnancy complications (10, 11), which increases the risk of adverse perinatal outcomes (12). Previous studies found that the risk of pregnancy-related complications and adverse pregnancy outcomes *via* ART was higher than *via* spontaneous conception (13–15), and a recent meta-analysis showed that patients with PCOS undergoing IVF were associated with higher risks of adverse pregnancy outcomes (16). However, studies on the association between various PCOS phenotypes after IVF/ICSI and adverse perinatal outcomes were relatively small.

The present study retrospectively analyzed the adverse perinatal outcomes of patients with various PCOS phenotypes who underwent IVF/ICSI.

## Materials and Methods

### Study Patients

We screened patients who underwent their first IVF/ICSI cycle at the Center for Reproductive Medicine, Cheeloo College of Medicine, Shandong University between January 2017 and July 2018. All patients were divided into the PCOS group and the control group. PCOS was defined according to the Rotterdam consensus criteria (2004) (17); that is, PCOS was diagnosed if at least two of the following criteria were present: oligomenorrhea/anovulation (defined as delaying of >35 days or <8 spontaneous hemorrhagic episodes/year), clinical and/or biochemical hyperandrogenism [biochemical hyperandrogenism was defined as total testosterone levels above 48.1 ng/dl detected in patients with no clinical evidence of hyperandrogenism or menstrual disturbances and not taking hormonal medication, and hirsutism was defined as patients with a total score ≥6 by the modified Ferriman–Gallwey score (18)], and polycystic ovary on ultrasonography (≥12 small follicles measuring 2–9 mm in at least one ovary and/or ovarian volume ≥10 cm^3^), and it is necessary to exclude other endocrine dysfunctions. Furthermore, the PCOS group was classified into four phenotype subgroups as follows (19): phenotype A—HA+OA+PCO, phenotype B—HA+OA, phenotype C—HA+PCO, and phenotype D—OA+PCO. Women in the control group had regular menstrual cycles (21–35 days), without evidence of HA or PCO. All patients with the following conditions were excluded: age >38 years old, serum FSH level >15 IU/L, diabetes, hypertension, abnormal parental karyotypes, severe intrauterine adhesion or uterine abnormality, chronic medical conditions that contraindicated pregnancy or with other endocrine dysfunction (such as Cushing’s syndrome, primary hyperprolactinemia, thyroid dysfunction, congenital adrenal hyperplasia, androgen producing neoplasm), and history of recurrent spontaneous abortion (RSA) or unilateral oophorectomy.

In total, we identified 6,732 women who met the study criteria, consisting of 1,186 in the PCOS group and 5,546 in the control group. This study was approved by the Institutional Review Board of the Center for Reproductive Medicine, Cheeloo College of Medicine, Shandong University (2017-53).

### Measurement

All patients underwent clinical history (including but not limited to the menstrual cycle and infertility type), physical examination [including but not limited to body mass index (BMI), Ferriman–Gallwey score, and gynecologic examination], biochemical analysis [including but not limited to the levels of fasting blood glucose (FBG), follicle-stimulating hormone (FSH), luteinizing hormone (LH), estradiol, progesterone, total testosterone (To), anti-Müllerian hormone (AMH) and thyroid-stimulating hormone (TSH), and prolactin], and transvaginal ultrasonography for calculated antral follicle count (AFC) on follicular phase. Blood samples were drawn for biochemical analyses on days 2–3 of a spontaneous or progestogen-induced menstrual cycle. All the hormonal assays were made at the Center for Reproductive Medicine Laboratory, Cheeloo College of Medicine, Shandong University.

### Treatment Protocol

According to a routine method (18), all patients received a standardized ovarian stimulation regimen; underwent oocyte retrieval, fertilization, and transfer embryos; and were provided luteal phase support. All patients underwent COH with standard long agonist protocol or antagonist protocol [as previously described (20, 21)]. As monitored on ultrasound and based on the level of serum sex hormones (including FSH, LH, E_2_, progesterone), Gn doses were adjusted based on the ovarian response. Human chorionic gonadotropin (HCG) at a dose of 4,000 to 8,000 IU was administered when at least two follicles were ≥18 mm. Oocyte retrieval was performed 34–36 h later under transvaginal ultrasound guidance. According to sperm quality, IVF/ICSI was performed. The embryo quality was graded according to the number of blastomeres, percent fragmentation, and regularity. Embryos were transferred on day 3 or day 5 after oocyte retrieval according to the patient’s condition (such as embryo quality, abdominal distention, and endocrine examination results). Cycle cancellation is defined if the patient does not have a fresh embryo transfer after oocyte retrieval (and we excluded cycles canceled before HCG triggering). Luteal phase support was provided after oocyte retrieval for those women who planned to transfer fresh embryos, as previously described (18, 20). Fourteen days after embryo transfer, the serum HCG levels were measured. If conception occurred, the luteal phase support was maintained. Transvaginal ultrasonography was performed 35 days after embryo transfer.

### IVF/ICSI Outcomes

In this study, the primary outcome measures were adverse perinatal outcomes, while the secondary outcome measures included biochemical pregnancy, clinical pregnancy (CP), and live birth (LB). Adverse perinatal outcomes were categorized into adverse pregnancy outcomes and pregnancy complications. Adverse pregnancy outcomes included ectopic pregnancy, miscarriage, and premature birth, and pregnancy complications included hypertensive disorders of pregnancy (HDP), gestational diabetes mellitus (GDM), and others (postpartum hemorrhage, placenta previa, placental abruption, premature rupture of membrane, cardiac diseases complicating pregnancy). Ectopic pregnancy was considered as developing blastocyst implanted outside the endometrial cavity. Miscarriage was defined as clinical pregnancy lost before 28 weeks of gestation. Premature birth was defined as a baby born between the 28th and 37th week of pregnancy. In this study, HDP included gestational hypertension (333 cases) and preeclampsia (1 case). Gestational hypertension and preeclampsia were defined as previously described (22–24). GDM was defined as the variable severity of glucose intolerance with onset or first recognition during pregnancy (25). Biochemical pregnancy was defined as serum HCG level ≥10 IU/L. CP was defined as the presence of gestational sacs by ultrasonography. LB was defined as the delivery of any viable infant at 28 weeks or more of gestation. Additionally, the cycle cancellation rate was calculated as the number of canceled fresh embryo transfer cycles divided by the number of oocyte retrieval cycles. Embryos of grades I and II, with 7–10 cells on day 3, were defined as high-quality embryos, and high-quality embryo rate, defined as the number of high-quality embryo/number of zygotes, was calculated. Fertilization rate (FR) was calculated as the number of 2PN divided by the number of oocyte retrieval, and implantation rate (IR) was calculated as the number of observed gestational sacs divided by the number of transferred embryos.

### Statistical Analysis

Comparisons between groups were performed using one-way analysis of variance (with the LSD *post-hoc* test) for continuous variables and the chi-squared test (or Fisher’s exact test when the expected frequencies were less than five) for categorical variables. The results were expressed as mean ± standard deviation (SD) for continuous variables and as percentages for categorical variables. The study was retrospective to balance basic patient characteristics (including age, infertility type, and stimulation protocol) between groups. We used 1:1 propensity score matching (PSM) to match control patients to PCOS patients, and 0 is the matching caliper of PSM in this study. In the PCOS subgroups, logistic regression was used to evaluate the relationship between PCOS phenotype and IVF/ICSI outcomes while adjusting for relevant confounders, and the results were expressed as odds ratios (OR) with 95% confidence intervals (CI).

All statistical analyses were performed using the Statistical Package for Social Sciences (version 26.0, SPSS Inc., Chicago, USA) and R software. *P*-value <0.05 was considered statistically significant.

## Results

**Figure 1:**
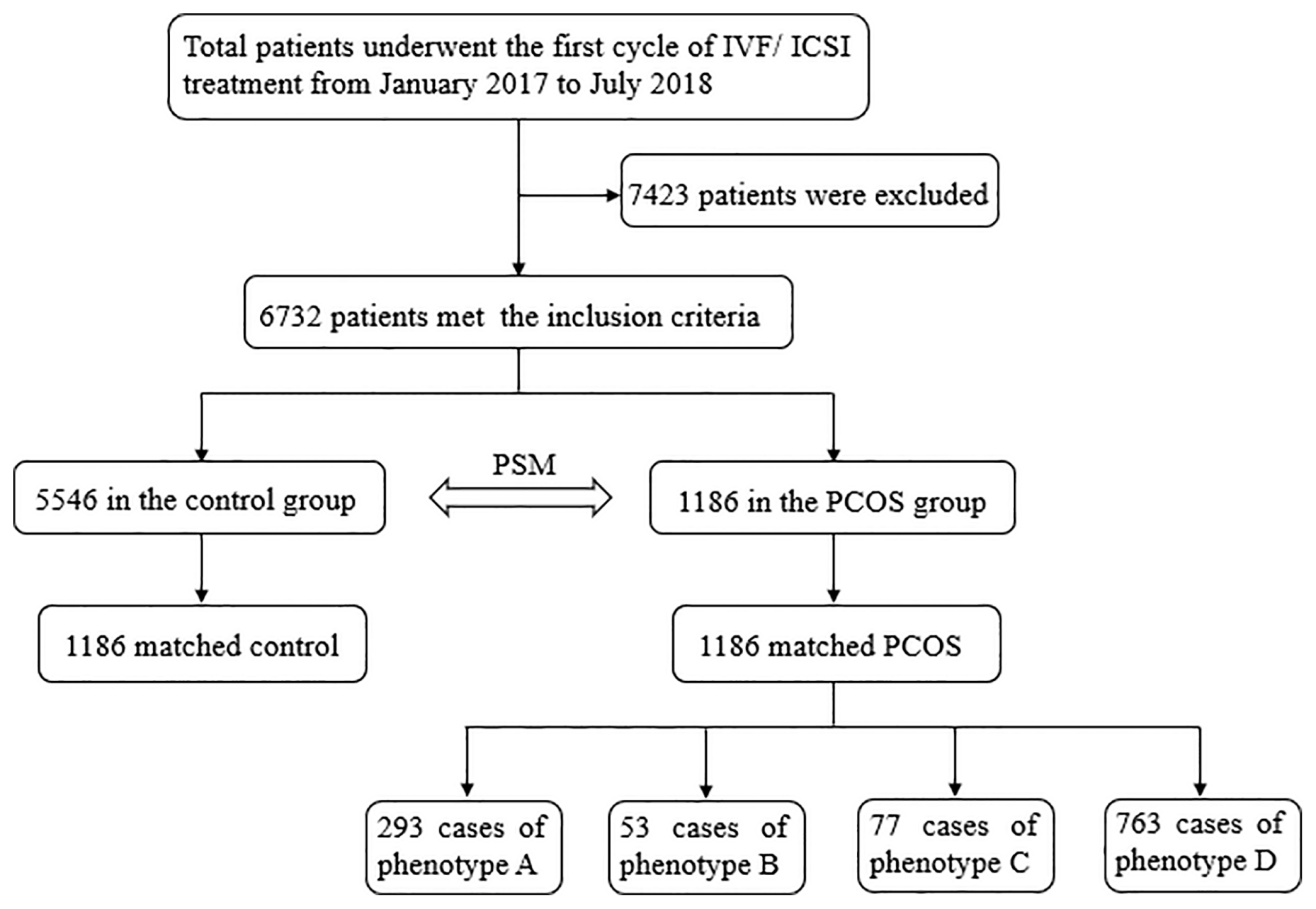
Flow chart database searching pathway and group divisions.

A total of 6,732 patients were recorded, with 1,186 in the PCOS group and 5,546 in the control group. After the PSM procedure, 1,186 patients were included in the control group, and there were 293 cases of phenotype A, 53 cases of phenotype B, 77 cases of phenotype C, and 763 cases of phenotype D in the PCOS groups (**Figure 1**).

### Patients’ Characteristics

The basic characteristics of the patients among the five groups are shown in **Table 1**. The results showed significant differences in BMI, FBG, FSH, LH, LH/FSH ratio, To, AMH, and AFC among the five groups (all *P* < 0.001). Of these, BMI, FBG, LH, and To were higher in the PCOS phenotype A group than in the other groups (all *P* < 0.001). The basic characteristics before PSM are shown in **Supplementary Table 1**. In addition, we only compared the 2-h plasma glucose concentrations after OGTT in various PCOS phenotype groups, and the results showed no statistically significant differences between groups (*P* = 0.633, data not shown).

**Table 1:** Basic characteristics of the patients among the four PCOS phenotype groups and the control group.

	Phenotype A (*n* = 293)	Phenotype B (*n* = 53)	Phenotype C (*n* = 77)	Phenotype D (*n* = 763)	Matched control (*n* = 1,186)	*P*-value*
Age (years)	28.92 ± 3.36	28.87 ± 3.02	28.90 ± 3.12	29.49 ± 3.46	29.28 ± 3.40	0.095
BMI (kg/m^2^)	25.79 ± 3.91^c^ ^e^	24.78 ± 3.25^e^	24.45 ± 3.75^a^ ^d^ ^e^	25.35 ± 3.88^c^ ^e^	23.43 ± 3.54^a^ ^b^,^c^ ^d^	<0.001
FBG (mmol/L)	5.37 ± 0.74^e^	5.22 ± 0.38	5.33 ± 0.41	5.32 ± 0.50^e^	5.22 ± 0.45^a^ ^d^	<0.001
FSH (IU/L)	5.71 ± 1.36^e^	6.08 ± 1.49^d^	5.88 ± 1.17^e^	5.57 ± 1.25^b^,^e^	6.37 ± 1.67^a^ ^c^ ^d^	<0.001
LH (IU/L)	11.68 ± 5.30^b^ ^c^ ^d^ ^e^	8.80 ± 4.67^a^ ^e^	8.34 ± 5.55^a^ ^e^	8.20 ± 4.94^a^ ^e^	5.20 ± 2.95^a^ ^b^ ^c^ ^d^	<0.001
LH/FSH	2.07 ± 0.89^b^ ^c^ ^d^ ^e^	1.49 ± 0.82^a^ ^e^	1.42 ± 0.86^a^ ^e^	1.48 ± 0.87^a^ ^e^	0.86 ± 0.58^a^ ^b^ ^c^ ^d^	<0.001
To (ng/dl)	62.28 ± 14.57^c^ ^d^ ^e^	60.00 ± 12.18^d^ ^e^	58.29 ± 11.31^a^ ^d^ ^e^	31.78 ± 10.15^a^ ^b^ ^c^ ^e^	25.04 ± 10.72^a^ ^b^ ^c^ ^d^	<0.001
AMH (ng/ml)	12.35 ± 6.03^b^ ^c^ ^d^ ^e^	7.05 ± 3.91^a^ ^c^ ^d^ ^e^	8.86 ± 4.37^a^ ^b^ ^e^	9.37 ± 4.80^a^ ^b^ ^e^	4.43 ± 3.05^a^ ^b^ ^c^ ^d^	<0.001
AFC	33.73 ± 11.64^b^ ^c^ ^d^ ^e^	16.70 ± 3.66^a^ ^c^ ^d^	27.74 ± 8.62^a^ ^b^ ^e^	28.98 ± 8.10^a^ ^b^ ^e^	15.33 ± 6.11^a^ ^c^ ^d^	<0.001
Infertility type, *n* (%)						0.222
Primary	176 (60.1)	35 (66.0)	57 (74.0)	468 (61.3)	737 (62.1)	
Secondary	117 (39.9)	18 (34.0)	20 (26.0)	295 (38.7)	449 (37.9)	

BMI, body mass index; FBG, fasting blood glucose; FSH, follicle-stimulating hormone; To, total testosterone concentration; AMH, anti-Müllerian hormone; AFC, antral follicle count.

a Significantly different from phenotype A.

b Significantly different from phenotype B.

c Significantly different from phenotype C.

d Significantly different from phenotype D.

e Significantly different from the control group.

^*^All P-values for quantitative variables were determined by post-hoc analysis (LSD).

### Ovarian Response and Pregnancy Outcomes

The ovarian response and pregnancy outcomes of patients among the four PCOS phenotype groups and the control group are presented in **Table 2**. Significant differences in HCG dose, endometrial thickness, the number of follicles of diameter ≥14 mm and E_2_ levels on the trigger day, the number of retrieved oocytes and frozen embryos, and high-quality embryo rate among groups were observed (all *P* < 0.05). Of these, the number of follicles of diameter ≥14 mm and E_2_ levels on the trigger day and the number of retrieved oocytes were significantly higher in PCOS phenotype A and C groups compared with the other phenotype groups and the control group (all *P* < 0.05). It is worth noting that the high-quality embryo rate of PCOS phenotype A and D groups was lower than that of the other groups, especially the control group (*P* = 0.019). Although there were significant differences in Gn priming dose, stimulation duration, and the number of 2PN among the five groups (all *P* < 0.001), the total dose of Gn and FR were not statistically different (all *P *> 0.05). We can see that the cycle cancellation rate of the PCOS phenotype D group is lower than that of PCOS phenotype A and C groups and higher than that of PCOS phenotype B and control groups (62.8% and 68.8% vs. 53.9% vs. 39.6% and 34.8%, *P* < 0.001). The patients in the five groups had similar biochemical pregnancy rates, CPRs, ectopic pregnancy rates, miscarriage rates, premature birth rates, and LBRs (all *P *> 0.05). The data on ovarian response and pregnancy outcomes before PSM are shown in **Supplementary Table 2**. In addition, we compared the incidence of ovarian hyperstimulation syndrome (OHSS) in various PCOS phenotype groups after IVF-ET, and the results showed no statistically significant differences between groups (*P* = 0.788, data not shown).

**Table 2:** Comparison of ovarian response and pregnancy outcomes among the four PCOS phenotype groups and the control group.

	Phenotype A (*n* = 293)	Phenotype B (*n* = 53)	Phenotype C (*n* = 77)	Phenotype D (*n* = 763)	Matched control (*n* = 1,186)	*P*-value*
Stimulation protocol, *n* (%)						0.386
Long agonist	121 (41.3)	26 (49.1)	41 (53.2)	332 (43.5)	520 (43.8)	
Antagonist	172 (58.7)	27 (50.9)	36 (46.8)	431 (56.5)	666 (56.2)	
Gn priming dose (IU)	140.49 ± 28.87^b^ ^e^	152.83 ± 36.50^a^	143.99 ± 33.91^e^	143.38 ± 30.53^e^	158.27 ± 45.32^a^ ^c^ ^d^	<0.001
Total dose of Gn (IU)	1,812.47 ± 947.69	1,834.67 ± 818.40	1,721.27 ± 887.01	1,827.65 ± 857.09	1,842.21 ± 758.18	0.781
Stimulation duration (days)	10.55 ± 2.52^e^	10.15 ± 2.17	10.04 ± 2.40	10.48 ± 2.39^e^	9.87 ± 1.81^a^ ^d^	<0.001
HCG dose (IU)	6,139.93 ± 1,756.85^b^ ^d^ ^e^	6,660.38 ± 1,640.16^a^ ^e^	6,272.73 ± 1,675.18^e^	6,570.12 ± 1,672.30^a^ ^e^	7,265.18 ± 1,502.00^a^ ^b^ ^c^ ^d^	<0.001
Endometrial thickness on the trigger day (mm)	10.43 ± 2.01^d^ ^e^	10.69 ± 2.62	10.67 ± 1.94	10.90 ± 1.92^a^	10.96 ± 1.95^a^	0.001
No. of follicles of diameter ≥14 mm on the trigger day	15.72 ± 6.04^b^ ^d^ ^e^	13.02 ± 4.85^a^ ^c^ ^d^ ^e^	16.26 ± 5.97^b^ ^d^ ^e^	14.56 ± 5.57^a^ ^b^ ^c^ ^e^	10.77 ± 4.73^a^ ^b^ ^c^ ^d^	<0.001
E_2_ levels on the trigger day (pg/ml)	4,882.83 ± 2,918.41^b^ ^d^ ^e^	4,161.73 ± 2,444.63^a^ ^e^	4,790.87 ± 2,823.48^d^ ^e^	4,168.88 ± 2,488.18^a^ ^c^ ^e^	3,233.50 ± 1,826.45^a^ ^b^ ^c^ ^d^	<0.001
No. of retrieved oocytes	15.82 ± 8.07^b^ ^d^ ^e^	12.60 ± 6.11^a^ ^c^ ^d^	16.17 ± 7.12^b^ ^d^ ^e^	14.56 ± 6.94^a^ ^b^ ^c^ ^e^	11.08 ± 5.45^a^ ^c^ ^d^	<0.001
No. of 2PN	8.44 ± 4.50^e^	7.98 ± 4.61	8.92 ± 3.85^e^	8.29 ± 3.98^e^	6.90 ± 3.78^a^ ^c^ ^d^	<0.001
FR (%)	0.61 ± 0.21^e^	0.65 ± 0.24	0.63 ± 0.21	0.64 ± 0.21	0.64 ± 0.23^a^	0.269
High-quality embryo rate (%)	34.20 ± 22.08^e^	38.16 ± 20.45	38.48 ± 21.72	35.75 ± 22.06^e^	38.45 ± 23.79^a^ ^d^	0.019
Cycle cancellation rate, *n* (%)	184/293 (62.8)^b^ ^d^ ^e^	21/53 (39.6)^a^ ^c^ ^d^	53/77 (68.8)^b^ ^d^ ^e^	411/763 (53.9)^a^ ^b^ ^c^ ^e^	413/1,186 (34.8)^a^ ^c^ ^d^	<0.001
No. of transferred embryos	1.72 ± 0.45^e^	1.72 ± 0.46^e^	1.71 ± 0.46	1.64 ± 0.48^e^	1.54 ± 0.50^a^ ^b^ ^d^	<0.001
No. of transferred high-quality embryos	1.69 ± 0.52^e^	1.72 ± 0.46^e^	1.67 ± 0.57	1.61 ± 0.53^e^	1.52 ± 0.53^a^ ^b^ ^d^	0.001
IR (%)	56.40 ± 44.67	57.80 ± 44.19	56.30 ± 44.99	55.00 ± 44.30^e^	48.30 ± 44.52^d^	0.077
Biochemical pregnancy rate/ET cycles (%)	84/109 (77.1)^e^	23/32 (71.9)	18/24 (75.0)	258/352 (73.3)^e^	521/773 (67.4)^a^ ^d^	0.123
CPR/ET cycles (%)	75/109 (68.8)^e^	22/32 (68.8)	16/24 (66.7)	233/352 (66.2)^e^	453/773 (58.6)^a^ ^d^	0.051
LBR (%)	59/109 (54.1)	17/32 (53.1)	15/24 (62.5)	190/352 (54.0)	386/773 (49.9)	0.543

Gn, gonadotropin; HCG, human chorionic gonadotropin; FR, fertilization rate; IR, implantation rate; CPR, clinical pregnancy rate; LBR, live birth rate.

a Significantly different from phenotype A.

b Significantly different from phenotype B.

c Significantly different from phenotype C.

d Significantly different from phenotype D.

e Significantly different from the control group.

^*^All P-values for quantitative variables were determined by post-hoc analysis (LSD).

### Adverse Perinatal Outcomes

The adverse perinatal outcomes of the five groups are displayed in **Table 3**. The adverse pregnancy outcome rate was higher in PCOS phenotype A and D groups than in the control group (44.0% and 46.4% vs. 28.7%, *P* = 0.027). Despite the differences in HDP rate of PCOS phenotype A and C groups and the control group (9.3% and 12.5% vs. 3.1%, *P* = 0.037), the incidence of total pregnancy complications, GDM, or other pregnancy complications was similar among the five groups. There was no difference between the groups for the rates of ectopic pregnancy, miscarriage, premature birth, cesarean section, and multiple births (all *P *> 0.05). The data on adverse perinatal outcomes before PSM are shown in **Supplementary Table 3**. In addition, the statistical power of the R×C square test was calculated *via* the “pwr” package in R software, where the effect size was determined as 0.55 using the ES.w2() function, and the statistical power was calculated as pwr.chisq.test(w = ES.w2(prob), *N* = 799, *df* = 4, sig.level = 0.05) >0.99, based on which we admit that the results in **Table 3** are accurate.

**Table 3:** Comparison of adverse perinatal outcomes among the four PCOS phenotype groups and the control group.

	Phenotype A (*n* = 75)	Phenotype B (*n* = 22)	Phenotype C (*n* = 16)	Phenotype D (*n* = 233)	Matched control (*n* = 453)	*P*-value^*^
Adverse pregnancy outcome rate (%)	33/75 (44.0)^e^	10/22 (45.5)	4/16 (25.0)	84/233 (46.4)^e^	130/453 (28.7)^a,d^	0.027
Ectopic pregnancy rate (%)	2/75 (2.7)	0/22 (0.0)	0/16 (0.0)	5/233 (2.1)	13/453 (2.9)	0.959
Miscarriage rate (%)	14/75 (18.7)	5/22 (22.7)	1/16 (6.3)	39/233 (16.7)	53/453 (11.7)	0.121
Premature birth rate (%)	17/75 (22.7)	5/22 (22.7)	3/16 (18.8)	40/233 (17.2)	64/453 (14.1)	0.266
Pregnancy complication rate (%)	13/75 (17.3)	4/22 (18.2)	2/16 (12.5)	40/233 (17.2)	57/453 (12.6)	0.429
HDP rate (%)	7/75 (9.3)^e^	1/22 (4.5)	2/16 (12.5)^e^	13/233 (5.6)	14/453 (3.1)^a,c^	0.037
GDM rate (%)	7/75 (9.3)	2/22 (9.1)	0/16 (0.0)	21/233 (9.0)	25/453 (5.5)	0.257
Rate of others (%)	0/75 (0.0)	1/22 (4.5)	0/16 (0.0)	7/233 (3.0)	19/453 (4.2)	0.343
Rate of cesarean section (%)	41/75 (54.7)	15/22 (68.2)	9/16 (56.3)	129/232 (55.6)	254/453 (56.1)	0.851
Multiple birth rate (%)	18/59 (30.5)	5/17 (29.4)	5/15 (33.3)	54/190 (28.4)	90/386 (23.3)	0.470

HDP, hypertensive disorders of pregnancy; GDM, gestational diabetes mellitus.

a Significantly different from phenotype A.

c Significantly different from phenotype C.

d Significantly different from phenotype D.

e Significantly different from the control group.

^*^All P-values for quantitative variables were determined by post-hoc analysis (LSD).

### Logistic Regression Assessment of Adverse Perinatal Outcomes

According to the previous results of adverse perinatal outcomes, a univariate logistic analysis of adverse pregnancy outcomes and HDP was performed. Compared with the control group, PCOS phenotypes A and D were the risk factors for adverse pregnancy outcomes [cOR (crude odds ratio)-A: 1.952, 95% CI-A: 1.185–3.216; cOR-D: 1.401, 95% CI-D: 1.001–1.960] and PCOS phenotype A was the risk factor for HDP (cOR: 3.228, 95% CI: 1.258–8.285). The factors with significant differences in the univariate analysis (these results are shown in **Supplementary Tables 4**, **5**) were included in the multivariate logistic regression analysis. After adjusting for confounding factors, PCOS phenotypes A and D were shown as independent risk factors for adverse pregnancy outcomes (aOR-A: 1.835, 95% CI-A: 1.095–3.075; aOR-D: 1.435, 95% CI-D: 1.025–2.008) (see **Table 4**).

**Table 4:** Logistic regression analysis of maternal and perinatal outcomes.

Outcomes	Phenotypes	cOR (95% CI)	*P*-value	aOR (95% CI)	*P*-value
Adverse pregnancy outcomes			0.030		0.025
	Control	Reference	–		–
	PCOS phenotype A	1.952 (1.185–3.216)	0.009	1.835 (1.095–3.075)	0.021
	PCOS phenotype B	2.071 (0.873–4.910)	0.099	2.084 (0.873–4.974)	0.098
	PCOS phenotype C	0.828 (0.262–2.615)	0.748	0.538 (0.169–1.720)	0.296
	PCOS phenotype D	1.401 (1.001–1.960)	0.049	1.435 (1.025–2.008)	0.035
HDP			0.085		0.898
	Control	Reference	–		–
	PCOS phenotype A	3.228 (1.258–8.285)	0.015	1.415 (0.337–5.944)	0.635
	PCOS phenotype B	1.493 (0.187–11.898)	0.705	0.807 (0.080–8.127)	0.856
	PCOS phenotype C	4.480 (0.928–21.624)	0.062	2.573 (0.436–15.185)	0.297
	PCOS phenotype D	1.853 (0.856–4.010)	0.117	1.385 (0.614–3.125)	0.433

cOR, crude odds ratio; CI, confidence interval; aOR, adjusted odds ratio.

The indicators with statistical differences in adverse pregnancy outcomes included patient type, BMI, To, HCG dose, E_2_ levels on the trigger day, no. of transferred embryos, and no. of transferred high-quality embryos, and the indicators with statistical differences in HDP included as patient type, BMI, and To.

## Discussion

In this study, the relationship between PCOS phenotypes and pregnancy was retrospectively analyzed in patients who underwent the first cycle of IVF/ICSI treatment. The results revealed that the PCOS phenotype was correlated with adverse pregnancy outcomes (ectopic pregnancy, miscarriage, and premature birth), and PCOS phenotypes A and D were the independent risk factors for adverse pregnancy outcomes. Moreover, CPR and LBR in various PCOS phenotypes were comparable.

Adverse pregnancy outcomes have been the subject of considerable attention, and the relationship between PCOS and adverse pregnancy outcomes has been a topic of great interest in the assisted reproductive field. A meta-analysis of pregnancy-related outcomes and complications in PCOS patients reported that PCOS patients present a high risk of adverse pregnancy outcomes despite the fact that they achieved a better LBR (16). Previous studies concluded that PCOS increased the risk of adverse pregnancy outcomes by affecting the reproductive endocrine and metabolic functions (6, 10, 26, 27). In addition, women with PCOS present with an abnormal endometrial phenotype and function (28), which possibly explains some of the adverse pregnancy outcomes such as miscarriage and premature birth (29).

The results of this study, for the first time, showed that PCOS phenotypes A and D were the independent risk factors for adverse pregnancy outcomes. In other words, higher incidences of adverse pregnancy outcomes occurred in women with PCOS phenotypes A and D. It was found that these two phenotypes of PCOS exist with common characteristics: OA and PCO. We speculated that the higher rates of adverse pregnancy in patients with PCOS result from a combined action of OA and PCO. A menstrual disorder in PCOS patients mainly results from insulin resistance, and it can reflect the degree of metabolic dysfunction (30). Recent findings showed that the menstrual patterns of PCOS patients might be correlated with the higher rates of adverse pregnancy outcomes (27). The result of a retrospective study showed that amenorrhea in PCOS patients was an independent risk factor for adverse pregnancy outcomes. Also, oocyte maturation and fertility rate in women with anovulation were lower than in women with regular cycling, and the development rate of the embryo shared a similar trend (31). Another study involving dairy cattle with anovulation reported that anovulation results in significant alterations in gene expression. Specifically, transcripts linked to the control of energy metabolism and DNA repair were downregulated, whereas genes involved in apoptosis and autophagy were upregulated. It was also found that the risk factors for OA have a direct impact on embryo development and endometrial receptivity (32).

Moreover, several studies suggested that PCO were associated with poor oocyte quality, and they also found elevated levels of homocysteine in the blood of PCOS patients (33–35) and in the follicular fluid of patients with PCO (36). These findings suggested that abnormally high homocysteine levels of follicular fluid were related to the poor quality of oocytes and low fertilization rates, even to the poor quality of embryos and adverse pregnancy outcomes (36). In a previous study, Jia et al. reported that the quality of oocytes in PCO has decreased, which could be due to mtDNA hypermethylation and abnormal activation of one-carbon metabolism (37). In addition, we also found that the high-quality embryo rate of PCOS phenotype A and D groups was lower than that of the other groups, especially the control group. This result supports our speculation. The coexistence of OA and PCO may be associated with higher rates of adverse pregnancy by affecting the quality of oocyte and embryo.

At present, advanced maternal age (38, 39), high levels of BMI (40, 41), and a thin endometrium (42–44) as risk factors for adverse pregnancy outcomes are well recognized in the literature. Therefore, multivariate logistic regression analyses in our study were performed to exclude the potential influences of these confounding factors, but the effect of PCOS phenotypes A and D on adverse pregnancy outcomes persists. In addition, a recent meta-analysis suggested that HA has adverse effects on assisted reproductive outcomes in patients with PCOS (45). However, the contribution of HA to miscarriage is still debated (46, 47). The effect of HA on adverse pregnancy outcomes was not found in our study, but the aOR of PCOS phenotype A (with HA) was higher than that of PCOS phenotype D (without HA) in the logistic analysis of adverse pregnancy outcomes. It was hypothesized that HA may have a role in the incidence of adverse pregnancy outcomes in IVF/ICSI and that this effect would be weak. Simultaneously, OA and PCO were the primary influencers in adverse pregnancy outcomes. As we all know, OHSS is also an important factor affecting adverse pregnancy outcomes (48), and patients with PCOS are at a greater risk to develop OHSS (49). In the present study, we compared the incidence of OHSS in various PCOS phenotype groups after IVF-ET, and the results showed no statistically significant differences between groups. These results were probably due to some PCOS patients with a higher OHSS risk canceling fresh embryo transfer and selecting all-embryo cryopreservation (50).

The results of our study highlight the need for individualized treatment and intensive follow-up after pregnancy in patients with PCOS phenotypes A and D, to decrease the incidence of adverse pregnancy outcomes. However, as with all retrospective data analyses, we were not able to completely rule out all potential confounders. Moreover, our study inevitably suffers from several limitations, even though we used PSM statistical methods to diminish bias. Although we have expanded the sample size compared with those reported in previous studies (8, 51), the sample size of some PCOS phenotypes is still the main limitation of the study. We think that one possible explanation could be the characteristics of the study population. Therefore, further prospective research with a sufficient sample size will be needed to confirm these findings in the future.

Taken together, our data revealed that PCOS phenotypes A and D were the independent risk factors for adverse pregnancy outcomes. Specifically, the higher incidences of adverse pregnancy outcomes occur in women with PCOS phenotypes A and D compared with women with non-PCOS. Therefore, for women with PCOS phenotypes A and D, individualized treatment during assisted reproduction and close follow-up after clinical pregnancy are necessary.

## Data Availability Statement

The raw data supporting the conclusions of this article will be made available by the authors, without undue reservation.

## Ethics Statement

The studies involving human participants were reviewed and approved by the Institutional Review Board of the Center for Reproductive Medicine, Cheeloo College of Medicine, Shandong University. The patients/participants provided their written informed consent to participate in this study.

## Author Contributions

YS, LY, and QW conceived and designed this study. QW contributed to the statistical analysis and interpretation of data and drafting of the manuscript. HW and PL performed the statistical analysis and participated in the discussion. XL and ZW analyzed and interpreted the data. LY and QW participated in the discussion and critically revised the manuscript. All authors read and approved the final manuscript.

## Funding

This study was supported by the National Key R&D Program of China (2021YFC2700404, 2018YFC1003202).

## Conflict of Interest

The authors declare that the research was conducted in the absence of any commercial or financial relationships that could be construed as a potential conflict of interest.

## Publisher’s Note

All claims expressed in this article are solely those of the authors and do not necessarily represent those of their affiliated organizations, or those of the publisher, the editors and the reviewers. Any product that may be evaluated in this article, or claim that may be made by its manufacturer, is not guaranteed or endorsed by the publisher.
