# Supplementary material for: Association of Polycystic Ovary Syndrome Phenotypes With Adverse Pregnancy Outcomes After In-Vitro Fertilization/Intracytoplasmic Sperm Injection
Source: Front Endocrinol (Lausanne). 2022 Jun 3;13:889029. doi: 10.3389/fendo.2022.889029 (PMC9203834; doi:10.3389/fendo.2022.889029)
Supplement: Supplementary file 1 [file Table_1.docx]

**Supplementary Table 1** Basic characteristics of the patients among four PCOS phenotype groups and control group before PSM

|  | Phenotype A(n=293) | Phenotype B(n=53) | Phenotype C(n=77) | Phenotype D(n=763) | Control  (n=5546) | *P*-value* |
| --- | --- | --- | --- | --- | --- | --- |
| Age (years) | 28.92±3.36^d,e^ | 28.87±3.02^e^ | 28.90±3.12^e^ | 29.49±3.46^a,e^ | 30.34±3.70^a,b,c,d^ | ＜0.001 |
| BMI (kg/㎡) | 25.79±3.91^c,e^ | 24.78±3.25^e^ | 24.45±3.75^a^ | 25.35±3.88^c,e^ | 23.67±3.52^a,b,d^ | ＜0.001 |
| FBG (mmol/l) | 5.37±0.74^e^ | 5.22±0.38 | 5.33±0.41 | 5.32±0.50^e^ | 5.23±0.49^a,d^ | ＜0.001 |
| FSH (IU/L） | 5.71±1.36^e^ | 6.08±1.49^d^ | 5.88±1.17^e^ | 5.57±1.25^b,e^ | 6.40±1.54^a,c,d^ | ＜0.001 |
| LH (IU/L) | 11.68±5.30^b,c,d,e^ | 8.80±4.67^a,e^ | 8.34±5.55^a,e^ | 8.20±4.94^a,e^ | 5.06±2.53^a,b,c,d^ | ＜0.001 |
| LH/FSH | 2.07±0.89^b,c,d,e^ | 1.49±0.82^a,e^ | 1.42±0.86^a,e^ | 1.48±0.87^a,e^ | 0.82±0.46^a,b,c,d^ | ＜0.001 |
| To (ng/dl) | 62.28±14.57^c,d,e^ | 60.00±12.18^d,e^ | 58.29±11.31^a,d,e^ | 31.78±10.15^a,b,c,e^ | 24.66±11.23^a,b,c,d^ | ＜0.001 |
| AMH (ng/ml) | 12.35±6.03^b,c,d,e^ | 7.05±3.91^a,c,d,e^ | 8.86±4.37^a,b,e^ | 9.37±4.80^a,b,e^ | 4.25±2.81^a,b,c,d^ | ＜0.001 |
| AFC | 33.73±11.64^a,b,c,e^ | 16.70±3.66^a,c,d^ | 27.74±8.62^a,b,e^ | 28.98±8.10^a,b,e^ | 15.20±5.84^a,c,d^ | ＜0.001 |
| Infertility type, n (%) |  |  |  |  |  | ＜0.001 |
| Primary | 176(60.1)^c,e^ | 35(66.0) | 57(74.0)^a,d,e^ | 468(61.3)^c,e^ | 2898(52.3)^a,c,d^ |  |
| Secondary | 117(39.9)^c,e^ | 18(34.0) | 20(26.0)^a,d,e^ | 295(38.7)^c,e^ | 2648(47.7)^a,c,d^ |  |

BMI=body mass index; FBG= fasting blood glucose; FSH=Follicle-Stimulating Hormone; To= total testosterone concentration; AMH=Anti-Müllerian hormone; AFC=antral follicle count

^a^ Significantly different from phenotype A

^b^ Significantly different from phenotype B

^c^ Significantly different from phenotype C

^d^ Significantly different from phenotype D

^e^ Significantly different from control group

^*^All *P*-values for quantitative variables were determined by post-hoc analysis (LSD)

**Supplementary Table 2** Comparison of ovarian response and pregnancy outcomes among four PCOS phenotype groups and control group before PSM

|  | | | Phenotype A(n=293) | | | Phenotype B(n=53) | Phenotype C(n=77) | | Phenotype D(n=763) | | Control  (n=5546) | *P*-value* |
| --- | --- | --- | --- | --- | --- | --- | --- | --- | --- | --- | --- | --- |
| Stimulation protocol, n (%) | | |  | | |  |  | |  | |  | ＜0.001 |
| Long agonist | | | 121(41.3)^e^ | | | 26(49.1)^e^ | 41(53.2)^e^ | | 332(43.5)^e^ | | 3895(70.2)^a,b,c,d^ |  |
| Antagonist | | | 172(58.7)^e^ | | | 27(50.9)^e^ | 36(46.8)^e^ | | 431(56.5)^e^ | | 1651(29.8) ^a,b,c,d^ |  |
| Gn priming dose (IU) | | | 140.49±28.87^e^ | | | 152.83±36.50 | 143.99±33.91^e^ | | 143.38±30.53^e^ | | 164.62±48.25^a,c,d^ | ＜0.001 |
| Total dose of Gn (IU) | | | 1812.47±947.69^e^ | | | 1834.67±818.40 | 1721.27±887.01^e^ | | 1827.65±857.09^e^ | | 1928.96±819.28^a,c,d^ | 0.001 |
| Stimulation duration (days) | | | 10.55±2.52^e^ | | | 10.15±2.17 | 10.04±2.40 | | 10.48±2.39^e^ | | 10.04±2.92^a,d^ | ＜0.001 |
| HCG dose (IU) | | | 6139.93±1756.85^b,d,e^ | | | 6660.38±1640.16^a,e^ | 6272.73±1675.18^e^ | | 6570.12±1672.30^a,e^ | | 7351.70±1426.36^a,b,c,d^ | ＜0.001 |
| Endometrial thickness on the trigger day (mm) | | | 10.4±2.0^d,e^ | | | 10.7±2.6 | 10.7±1.9 | | 10.9±1.9^a,e^ | | 11.1±2.0^a,d^ | ＜0.001 |
| No. of follicles of diameter ≥14mm on the trigger day | | | 15.72±6.04^b,d,e^ | | | 13.02±4.85^a,c,d,e^ | 16.26±5.97^b,d,e^ | | 14.56±5.57^a,b,c,e^ | | 10.99±4.67^a,b,c,d^ | ＜0.001 |
| E_2_ levels on the trigger day (pg/mL) | | | 4882.83±2918.41^b,d,e^ | | | 4161.73±2444.63^a,e^ | 4790.87±2823.48^d,e^ | | 4168.88±2488.18^a,c,e^ | | 3320.37±1840.46^a,b,c,d^ | ＜0.001 |
| No. of retrieved oocytes | | | 15.82±8.07^b,d,e^ | | | 12.60±6.11^a,c,d^ | 16.17±7.12^b,d,e^ | | 14.56±6.94^a,b,c,e^ | | 11.19±5.43^a,c,d^ | ＜0.001 |
| No. of 2PN | | 8.44±4.50^e^ | | | 7.98±4.61 | | 8.92±3.85^e^ | 8.29±3.98^e^ | | 7.03±3.82^a,c,d^ | | ＜0.001 |
| FR (%) | 0.61±0.21^e^ | | | 0.65±0.24 | | | 0.63±0.21 | | 0.64±0.21 | | 0.65±0.22^a^ | 0.022 |
| The percentage of quality embryos (%) | | | 34.20±22.08^e^ | | | 38.17±20.45 | 38.48±21.72 | | 35.75±22.06^e^ | | 38.99±23.57^a,d^ | 0.013 |
| Cycles cancellation rate, n (%) | | | 184/293(62.8)^b,d,e^ | | | 21/53(39.6)^a,c,d^ | 53/77(68.8)^b,d,e^ | | 411/763(53.9)^a,b,c,e^ | | 1816/5546(32.7)^a,c,d^ | ＜0.001 |
| No. of transferred embryos | | | 1.73±0.45^e^ | | | 1.72±0.46 | 1.71±0.46 | | 1.64±0.48 | | 1.61±0.49^a^ | 0.069 |
| No. of transferred good quality embryos | | | 1.69±0.52^e^ | | | 1.72±0.46 | 1.67±0.56 | | 1.61±0c.53 | | 1.58±0.54^a^ | 0.091 |
| IR | | | 0.56±0.45 | | | 0.58±0.44 | 0.56±0.45 | | 0.55±0.44^e^ | | 0.49±0.44^d^ | 0.047 |
| biochemical pregnancy rate/ET cycles (%) | | | 84/109(77.1) | | | 23/32(71.9) | 18/24(75.0) | | 258/352(73.3) | | 2577/3730(69.1) | 0.198 |
| CPR/ET cycles (%) | | | 75/109(68.8) | | | 22/32(68.8) | 16/24(66.7) | | 233/352(66.2) | | 2265/3730(60.7) | 0.104 |
| LBR/ET cycles（%） | | | 59/109(54.1) | | | 17/32(53.1) | 15/24(62.5) | | 190/352(54.0) | | 1914/3730(51.3) | 0.673 |

Gn= gonadotropin; HCG= human chorionic gonadotropin; FR= fertilization rate; IR= Implantation rate; CPR=clinical pregnancy rate; LBR= live birth rate

^a^ Significantly different from phenotype A

^b^ Significantly different from phenotype B

^c^ Significantly different from phenotype C

^d^ Significantly different from phenotype D

^e^ Significantly different from control group

^*^All *P*-values for quantitative variables were determined by post-hoc analysis (LSD)

**Supplementary Table 3** Comparison of adverse perinatal outcomes among four PCOS phenotype groups and control group before PSM

|  | | Phenotype A(n=293) | | | Phenotype B(n=53) | | Phenotype C(n=77) | | | Phenotype D(n=763) | | | Control  (n=5546) | | *P*-value^*^ | |
| --- | --- | --- | --- | --- | --- | --- | --- | --- | --- | --- | --- | --- | --- | --- | --- | --- |
| Adverse pregnancy outcomes rate (%) | | | | 27/75(36.0)^e^ | 8/22(36.4) | | | 2/16(12.5) | | | | 72/233(30.9) | | 582/2265 (25.7)^a^ | 0.055 |  |
| Ectopic pregnancy rate (%) | | 2/75 (2.7) | | | 0/22(0.0) | | | 0/16(0.0) | | 5/233 (2.1) | | 39/2265 (1.7) | | 0.732 |  |  |
| Miscarriage rate (%) | | 14/75(18.7) | | | 5/22(22.7) | | | 1/16(6.3) | | 39/233(16.7) | | 303/2265(13.4) | | 0.201 |  |  |
| Premature birth rate (%) | | 17/75(22.7) | | | 5/22(22.7) | | | 3/16(18.8) | | 40/233(17.2) | | 327/2265(14.4) | | 0.147 |  |  |
| Pregnancy complications rate (%) | | | | 13/75(17.3) | 4/22(18.2) | | | 2/16(12.5) | | | | 40/233(17.2) | 279/2265 (12.3) | | 0.136 |  |
| HDP rate (%) | | | 7/75(9.3) | 1/22(4.5) | | | 2/16(12.5) | | | | 13/233(5.6) | 98/2265 (4.3) | | 0.089 |  |  |
| GDM rate (%) | | | 7/75(9.3) | 2/22(9.1) | | | 0/16(0.0) | | | | 21/233(9.0) | 124/2265 (5.5) | | 0.087 |  |  |
| Rate of others (%) | | | 0/75(0.0) | 1/22(4.5) | | | 0/16(0.0) | | | | 8/233(3.4) | 73/2265 (3.2) | | 0.473 |  |  |
| Rate of cesarean section (%) | | 41/59(69.5) | | | 15/17(88.2) | | 9/15(60.0) | | | 129/189(68.3) | | | 1371/1923(71.3) | | 0.372 | |
| Multiple birth rate (%) | | 18/59(30.5) | | | 5/17(29.4) | | 5/15(33.3) | | | 54/190(28.4) | | | 507/1914(26.5) | | 0.835 | |

HDP= hypertensive disorders of pregnancy; GDM= gestational diabetes mellitus;

^a^ Significantly different from phenotype A

^b^ Significantly different from phenotype B

^c^ Significantly different from phenotype C

^d^ Significantly different from phenotype D

^e^ Significantly different from control group

^*^All *P*-values for quantitative variables were determined by post-hoc analysis (LSD)

**Supplementary Table 4** Comparison of patients (with normal or adverse pregnancy outcomes)’ basic characteristics and ovulation induction information

| Indicators | normal pregnancy  (n=538) | adverse pregnancy outcomes  (n=261) | P-value |
| --- | --- | --- | --- |
| Patient type, n (%) |  |  | 0.027 |
| Control | 323/538 (60.0) | 130/261 (49.8) |  |
| PCOS phenotype A | 42/538 (7.8) | 33/261 (12.6) |  |
| PCOS phenotype B | 12/538 (2.2) | 10/261 (3.8) |  |
| PCOS phenotype C | 12/538 (2.2) | 4/261 (1.5) |  |
| PCOS phenotype D | 149/538 (27.7) | 84/261 (32.2) |  |
| Age (years) | 29.22±3.33 | 29.64±3.48 | 0.102 |
| BMI (kg/㎡) | 24.35±3.77 | 25.08±4.12 | 0.016 |
| FBG (mmol/l) | 5.28±0.51 | 5.28±0.50 | 0.954 |
| FSH (IU/L） | 6.24±1.57 | 6.07±1.46 | 0.138 |
| LH (IU/L) | 6.62±4.57 | 6.84±4.14 | 0.512 |
| LH/FSH | 1.10±0.78 | 1.15±0.69 | 0.326 |
| To (ng/dl) | 30.22±15.46 | 34.06±17.02 | 0.001 |
| AMH (ng/ml) | 5.90±4.12 | 6.49±4.75 | 0.090 |
| AFC | 20.04±9.23 | 21.33±10.28 | 0.075 |
| Infertility type, n (%) |  |  | 0.213 |
| Primary | 343/538 (63.8) | 154/261 (59.0) |  |
| Secondary | 195/538 (36.2) | 107/261 (41.0) |  |
| Stimulation protocol, n (%) |  |  | 0.451 |
| Long agonist | 266/538 (49.4） | 137/261 (52.5) |  |
| Antagonist | 272/538 (50.6) | 124/261 (47.5) |  |
| Gn priming dose (IU) | 153.67±40.50 | 152.40±35.34 | 0.664 |
| Total dose of Gn (IU) | 1825.16±828.78 | 1887.07±762.23 | 0.310 |
| Stimulation duration (days) | 10.03±2.09 | 10.20±2.18 | 0.290 |
| HCG dose (IU) | 7508.36±1306.92 | 7704.98±1222.05 | 0.038 |
| Endometrial thickness on the trigger day (mm) | 11.11±1.82 | 11.04±2.00 | 0.634 |
| No. of follicles of diameter ≥14mm on the trigger day | 10.83±4.16 | 10.52±3.93 | 0.304 |
| E_2_ levels on the trigger day (pg/mL) | 2881.55±1323.51 | 2643.44±1096.27 | 0.007 |
| No. of retrieved oocytes | 10.25±3.98 | 9.96±3.74 | 0.326 |
| No. of 2PN | 6.77±3.33 | 6.55±3.23 | 0.373 |
| The percentage of quality embryos (%) | 0.40±0.22 | 0.40±0.23 | 0.884 |
| No. of transferred embryos | 1.60±0.49 | 1.70±0.46 | 0.008 |
| No. of transferred good quality embryos | 1.58±0.51 | 1.67±0.52 | 0.023 |

Note: Of these, there are the indicators with statistical differences in adverse pregnancy outcomes, such as patient type, BMI, To, HCG dose, E2 levels on the trigger day, No. of transferred embryos and No. of transferred good quality embryos.

**Supplementary Table 5** Comparison of patients (with non-HDP or HDP)’ basic characteristics and ovulation induction information

| Indicators | Non-HDP  (n=762) | HDP  (n=37) | P-value |
| --- | --- | --- | --- |
| Patient type, n (%) |  |  | 0.037 |
| Control | 439/762 (57.6) | 14/37 (37.8) |  |
| PCOS phenotype A | 68/762 (8.9) | 7/37 (18.9) |  |
| PCOS phenotype B | 21/762 (2.8) | 1/37 (2.7) |  |
| PCOS phenotype C | 14/762 (1.8) | 2/37 (5.4) |  |
| PCOS phenotype D | 220/762 (28.9) | 13/37 (35.1) |  |
| Age (years) | 29.34±3.39 | 29.68±3.15 | 0.560 |
| BMI (kg/㎡) | 24.50±3.87 | 26.36±4.12 | 0.005 |
| FBG (mmol/l) | 5.27±0.50 | 5.40±0.61 | 0.131 |
| FSH (IU/L） | 6.18±1.54 | 6.15±1.55 | 0.884 |
| LH (IU/L) | 6.68±4.45 | 6.84±4.17 | 0.838 |
| LH/FSH | 1.11±0.71 | 1.29±1.31 | 0.393 |
| To (ng/dl) | 31.12±15.98 | 38.79±16.51 | 0.005 |
| AMH (ng/ml) | 6.04±4.35 | 7.17±3.89 | 0.124 |
| AFC | 20.32±9.60 | 23.27±9.16 | 0.068 |
| Infertility type, n (%) |  |  | 0.386 |
| Primary | 471/762 (61.8) | 26/37 (70.3) |  |
| Secondary | 291/762 (38.2) | 117/37 (29.7) |  |
| Stimulation protocol, n (%) |  |  | 0.403 |
| Long agonist | 387/762 (50.8) | 16/37 (43.2) |  |
| Antagonist | 375/762 (49.2） | 21/37 (56.8) |  |
| Gn priming dose (IU) | 153.17±39.13 | 155.07±33.65 | 0.772 |
| Total dose of Gn (IU) | 1829.79±790.18 | 2166.55±1072.71 | 0.067 |
| Stimulation duration (days) | 10.05±2.09 | 10.86±2.58 | 0.067 |
| HCG dose (IU) | 7562.34±1286.89 | 7783.78±1181.69 | 0.305 |
| Endometrial thickness on the trigger day (mm) | 11.09±1.88 | 11.00±1.97 | 0.772 |
| No. of follicles of diameter ≥14mm on the trigger day | 10.71±4.06 | 11.24±4.62 | 0.435 |
| E_2_ levels on the trigger day (pg/mL) | 2817.02±1252.56 | 2530.87±1356.54 | 0.177 |
| No. of retrieved oocytes | 10.19±3.92 | 9.32±3.36 | 0.186 |
| No. of 2PN | 6.74±3.32 | 5.86±2.55 | 0.051 |
| The percentage of quality embryos (%) | 0.40±0.22 | 0.37±0.23 | 0.394 |
| No. of transferred embryos | 1.64±0.48 | 1.59±0.50 | 0.617 |
| No. of transferred good quality embryos | 1.61±0.51 | 1.57±0.56 | 0.600 |

Note: Of these, there are the indicators with statistical differences in HDP, such as patient type, BMI and To.
